# Susceptibility of Moroccan sheep and goat breeds to peste des petits ruminants virus

**DOI:** 10.1186/s13028-017-0323-y

**Published:** 2017-09-07

**Authors:** Fatima Zohra Fakri, Asmaa Elhajjam, Zahra Bamouh, Mohammed Jazouli, Zineb Boumart, Khalid Tadlaoui, Ouafaa Fassi-Fihri, Mehdi Elharrak

**Affiliations:** 1 Research and Development, MCI Santé Animale, Lot. 157, Z I, Sud-Ouest (ERAC), B.P. 278, 28810 Mohammedia, Morocco; 2 Institut Agronomique et Vétérinaire Hassan II, Rabat, Morocco

**Keywords:** Goat breed, Morocco, Peste des petits ruminants, Sheep breed, Susceptibility

## Abstract

**Background:**

Peste des petits ruminants (PPR) is a highly contagious viral disease of small ruminants in Asia and Africa. In 2008, a PPR outbreak was reported for the first time in Morocco and a mass vaccination campaign allowed control of the disease. In this study, the susceptibility of four Moroccan local breeds of small ruminants to PPR virus was investigated by experimental infections. The objective was to make recommendations for improved epidemiological surveillance in Morocco by evaluating the susceptibility of the dominant Moroccan small ruminant breeds. Three parameters were studied: hyperthermia, clinical scoring and virus excretion. The outcome was compared to Alpine goats, which are considered one of the most sensitive breeds.

**Results:**

The study showed that the local goat breed was the most sensitive breed with a susceptibility rate of 67%, followed by Timahdit, Beni Guil and Sardi sheep with 48, 29 and 26%, respectively. Serological testing including enzyme-linked immunosorbent assay and viral neutralization showed that the Timahdit breed developed a stronger antibody response compared to the other breeds. Although the clinical signs observed in the sheep were mild, evidence of viral excretion was detected by means of a polymerase chain reaction assay.

**Conclusions:**

It is recommended that effective surveillance should focus on susceptible breeds complemented with serological surveillance of the sheep population.

## Background

Peste des petits ruminants virus (PPRV) belongs to the genus *Morbillivirus* in the *Paramyxoviridae* family and is the cause of peste des petits ruminants (PPR), one of the most contagious diseases of small ruminants throughout Africa, Asia and Middle Eastern countries up to Turkey. PPR isone of the major limitations of small ruminant farming in these regions [1–3].

The Food and Agriculture Organization of the United Nations (FAO) [4, 5] and the World Organization for Animal Health (OIE) have implemented a global eradication program by prioritizing epidemiological surveillance, early case finding and comprehensive vaccination campaigns. Effective surveillance is based on knowledge of the disease and clinical sign in different ruminant species and breeds in a specific region. Subclinical infections in some animals may result in a carrier status making an eradication program difficult to implement successfully.

In this study, we investigated the susceptibility of three local Moroccan sheep breeds and a goat breed by experimental infection with PPRV. These local breeds represent 79% of the Moroccan sheep population and 64% of goats in Morocco. Timahdit sheep (TM) are found in the Atlas Mountains, Beni Guil sheep (BG) in the Oriental region and Sardi sheep (SD) in the Atlantic littoral zone. These represent 33, 24 and 22% of the total Moroccan sheep population, respectively. This is the first time breed susceptibility is tested by experimental challenge with PPRV.

The objective of this study was to determine the susceptibility of local Moroccan sheep and goat breeds to PPRV to improve the epidemiological surveillance in Morocco.

## Methods

### Animals

The experiment was conducted in a biosafety level 3 (BSL3) animal containment unit of MCI Santé Animale, Morocco. Three groups of five sheep of the TM, BG and SD breeds were included together with one group of six goats of the local goat breed (aged 8–12 months)and one group consisting of two Alpine goats (aged 5 months). All animals tested negative for PPRV antibodies by means of virus neutralization (VN) and enzyme-linked immunosorbent assay (ELISA). The animals were kept in quarantine 15 days before the start of the experiment.

### Virus strain

The PPR virulent strain MOR08 (HQ131927), belonging to lineage IV, was used for the experimental infection. This strain is routinely used in our laboratory for vaccine potency testing; it was originally isolated during the 2008 outbreak in Morocco from a sheep showing characteristic clinical signs of PPR.

### Experimental infection, sampling and monitoring

The animal experiments were carried out in accordance with guidelines for the care and handling of experimental animals compiled by the Committee for the Control and Supervision of Experiments on Animals.

Animals were infected by intravenous (IV) injection (2 mL) and intra-nasal (IN) spraying (2 mL) of the virulent strain according to the protocol of Elharrak et al. [6] using a virus titer of 10^5.4^ TCID_50_/mL.

Monitoring was based on daily observations for hyperthermia and clinical scoring according to Elharrak et al. [6] and Hammouchi et al. [7]. The baseline rectal temperature was established individually and at the group level during the quarantine period by repeated measurements (n = 30) and measured daily throughout the study. Hyperthermia was defined as a rectal temperature above 39 °C. Clinical scores were used to evaluate the severity of clinical signs and to allow comparison between animals and groups. A clinical scoring system was followed with a ranking from 0 to 4 based on the severity of: general clinical appearance, hyperthermia, alimentation, behavior, diarrhea, nasal discharge, salivation, respiratory symptoms including dyspnoea, coughing and sneezing. A total cumulative score of the assessed signs per animal per day were then calculated. Animals were euthanized if the cumulative clinical score reached a value between 15 and 18 but with particular emphasize on the severity dyspnea and diarrhea. All surviving animals were euthanized at the end of the study (35 days post infection) by IV injection of an overdose of pentobarbital.

Rectal, conjunctival and oro-pharyngeal swabs were taken every second day and analyzed by quantitative real-time reverse transcriptase-polymerase chain reaction (RT-qPCR) assay to monitor viral excretion. Blood samples were collected weekly for serological testing. Post mortem samples were taken from the lungs, mesenteric and pulmonary lymph nodes, trachea and liver for viral nucleic acid detection by RT-qPCR.

Evaluation of the susceptibility of each breed was based on three criteria: the number of days of hyperthermia in each group and the average per group; the clinical scoring per group and its average; and the viral excretion expressed by the number of positive swabs detected per group and the average.

The susceptibility is represented by the cumulative value averages for each group. The Alpine goats, which are considered as completely sensitive to the virus was used as a positive control.

### Antibody and virus detection

Serological testing was carried out using the VN test as described in the OIE Terrestrial Manual (Chapters 2.7.11 and 2.7.14). The ELISA was also used to detect PPRV antibody response using the kit ID Screen^®^ PPR Competition (PPRC-4P ID-VET, ID.vet, Grabel, France).

For viral nucleic acid detection, real-time RT-qPCR was used [8]. RNA extraction was performed using isolate genomic RNA Mini kit (BIO-52075, Bioline) and amplification with the “Kit superscript Tm III Platinum R one step qRt-PCR system” 11745-100, Invitrogen).

### Statistical analysis

Differences among groups regarding hyperthermia, clinical scoring and viral excretion were determined using a one-way analysis of variance (ANOVA) followed by Student’s *t* test. A P value of ≤0.05 was considered statistically significant.

## Results

### Hyperthermia

Sheep of the BG and SD breeds developed hyperthermia for 22 and 20 days, respectively, with an average maximum of 39.8 and 40.0 °C, respectively. Sheep of the TM breed and the local goat breed developed hyperthermia for 36 days (average maximum: 40.8 °C) and 68 days (average maximum 41.0 °C), respectively. The two Alpine goats had hyperthermia for totally 17 days (average maximum 41.0 °C). The highest average value of days with hyperthermia per group occurred in the local goat breed (11.3 days) followed by the TM sheep (7.2 days) (Table 1).

**Table 1:** Data on of sheep and goats exposed to peste des petits ruminants virus

Species	Breed	No of animals	Total days of hyperthermia per group	Average hyperthermia days per animal	Total CS per group	Average of CS per animal	Total of positive swabs with PCR assays	Average of positive swabs	Sensitivity value	Percentage of susceptibility
Sheep	SD	5	20	4	9	1.8	10	2	7.8	25.8
	TM	5	36	7.2	14	2.8	26	5.2	15.2	47.6
	BG	5	22	4.4	9	1.8	14	2.8	9	29.4
Goats	Alpine	2	17^a^	8.5	37^a^	18.5	12^a^	6^a^	33	100
	Local	6	68^a^	11.3	53^a^	8.8	11^a^	1.8^a^	21.9	67.4

*CS* clinical scoring, *TM* Timahdit sheep, *BG* Beni Guil sheep, *SD* Sardi sheep

^a^The cumulative values are not directly comparable with the other groups due to another group size

The rectal temperature increase was significantly higher for the TM breed than for the BG and SD sheep (P ≤ 0.05), while these two breeds had a similar increase (P ≥ 0.05). The local goat breed had an increase in rectal temperature that was significantly higher than for any of the other groups.

### Clinical scoring

**Fig. 1:**
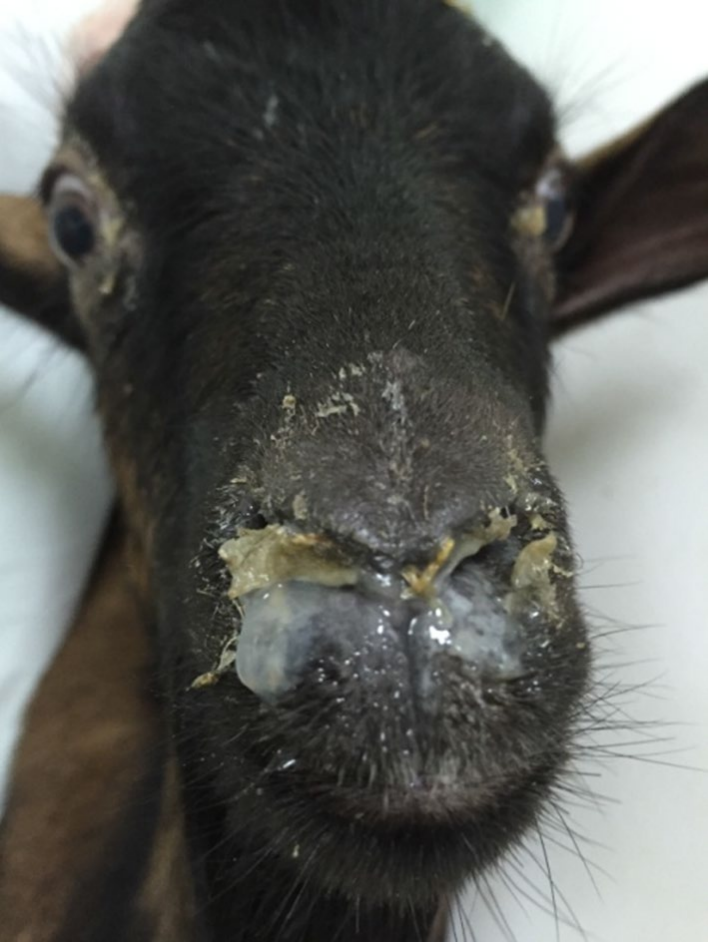
Nasal discharge observed on peste des petits ruminants virus infected Alpine goat

Mild clinical signs were observed in the BG and SD sheep, an intermediate level in the TM sheep and severe clinical disease in Alpine and local goats (Fig. 1). The clinical scoring and the average for each group are shown in Table 1. The cumulative clinical scoring was similar for the BG and SD sheep (P ≥ 0.05), whereas the clinical scoring was significantly higher for the goats irrespectively of breed (P < 0.05) and significantly more severe in the Alpine goats than in the goats of local origin (P ≤ 0.05).

Two Alpine goats died unexpected at day 29 with a clinical score of 18 and 19, respectively while three goats of the local breed died unexpected on days 12, 29 and 33 respectively, with a clinical score of 9, 10 and 10. In spite of the high score reached by the Alpine goats (cumulative effect), severity of the clinical signs was not sufficient to justify euthanasia.

**Fig. 2:**
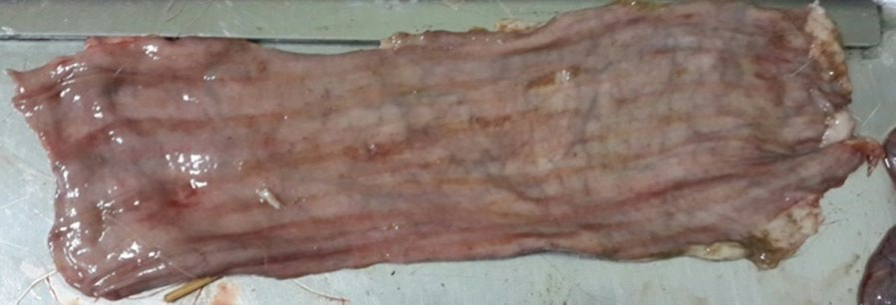
Post mortem intestine of Alpine goat with characteristic lesions characteristic linear red areas of congestion (“Zebra stripes”)

Post mortem gross lesions consisted of minor erosions and petechiae in the tracheal mucosa, bronchopneumonia, enlargement of lymph nodes, especially the mesenteric nodes, moderate mucosal congestion of the small intestine and colon with characteristic Zebra-like stripes (Fig. 2). None of the sheep died during the study but were euthanized at the end of the study. Post mortem examination revealed bronchopneumonia and moderate reaction of intestine nodes.

### Viral excretion

A high number of positive swabs (n = 12) were obtained from the Alpine goats with threshold values (Ct) between 28 and 32 during 3 days, and for the TM sheep, Ct values between 30 and 34 during 8 days. Lower excretion was reported for goats of the local origin (Ct: 29–35) during 2 days, BG and SD sheep (Ct: 35–37 and 32–36, respectively) during 4 days. Numbers of positive swabs are reported in Table 1. Regarding the average values of positive swabs, the highest number was recorded for TM sheep and Alpine goats, while there were no significant differences between local goats and BG and SD sheep. The viral excretion was detected beginning from day 2 to day 4 after infection, with no significant difference between breeds.

### Serology

**Fig. 3:**
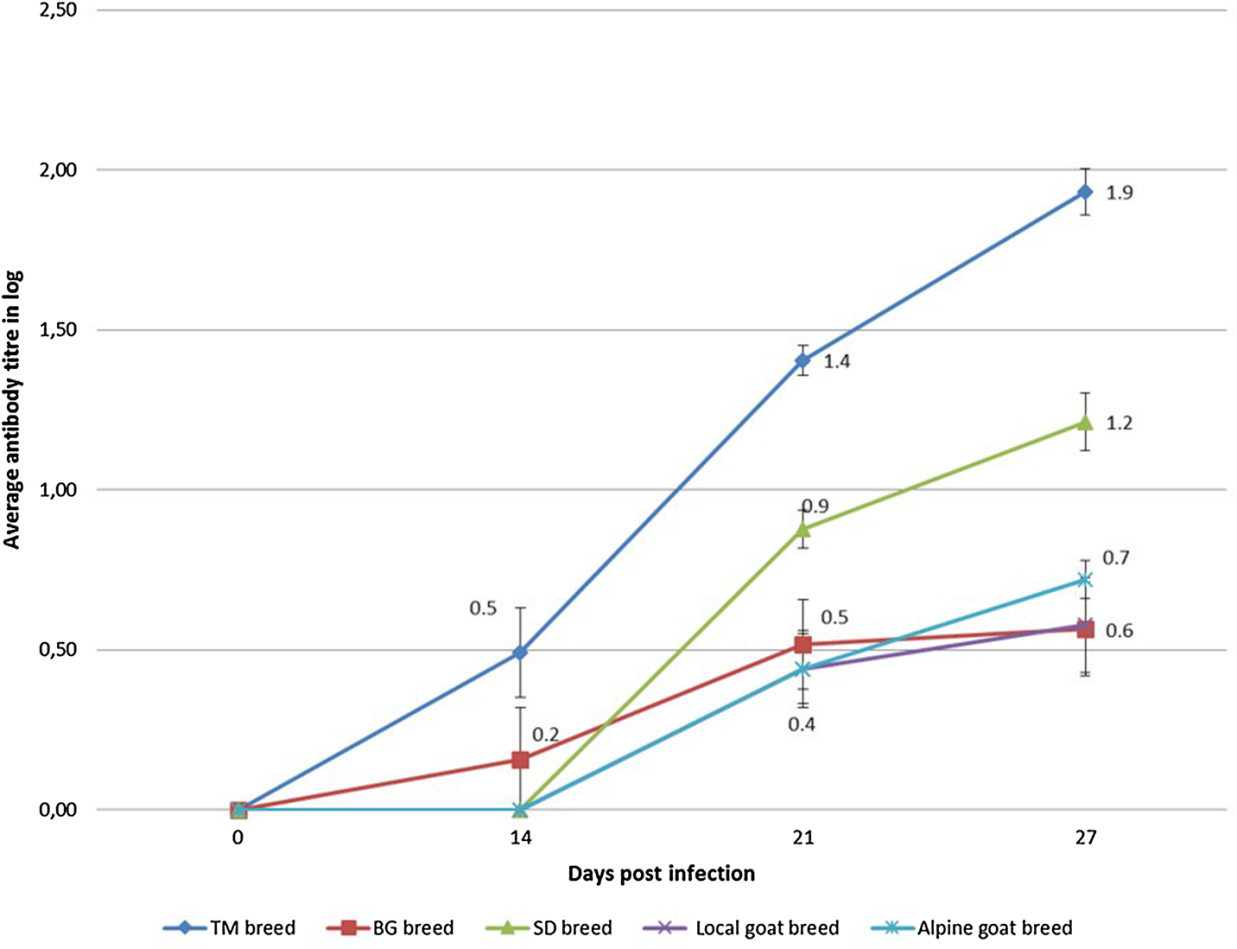
Neutralising peste des petits ruminants virus antibody response after experimental infection of sheep and goats. *TM* Timahdit sheep, *BG* Beni Guil sheep, *SD* Sardi sheep

Antibody responses were observed in animals of all groups from day 14 post infection to day 27. Sheep developed higher titers than goats (Fig. 3).

## Discussion

Goats appear to be very sensitive to the PPRV and develop characteristic and severe clinical signs associated with a high mortality rate [9]. Within African goat breeds, the Guinean breeds West African dwarf, Iogoon, Kindi and Djallonke are highly susceptible [10–12], while sheep have a milder course of disease [13–16].

In North Africa, there are approximately 70 million sheep and 20 million goats. During the 2008 PPR outbreak in Morocco, characteristic signs of the disease were observed only in young animals of some local sheep and goat breeds. The same observation was made in Algeria and Tunisia, where 5 years of serological surveillance has revealed a high prevalence of PPRV exposure [17–19]. Resistant animals are potential carriers of PPRV and contribute to maintain the infection as enzootic in a region.

In this study, experimental infection using a virulent strain of PPRV isolated during the 2008 outbreak in Morocco did cause severe disease in goats but only mild clinical signs in sheep. The greatest susceptibility was registered in local goats followed by TM sheep, BG sheep and finally SD sheep (Table 1). The findings were compared to those of Alpine goats for which susceptibility is considered as 100%. Other studies have reported death in Alpine goats 8–12 days post infection [6, 7], while in the present study deaths occurred 4 weeks after inoculation. This may be due to the different virus titer used. Also, a long storage of the challenge strain may have affected its virulence.

Local goats seems less susceptible to PPRV than Alpine goats, however, during the natural spread of the virus in Morocco, no special mention of morbidity in goats was reported. It is probably due to the specific goat breeding system, which is extensive in large areas with limited veterinary services.

The results confirmed what was observed on the field during the emergence of the 2008 outbreak. Indeed, very few cases have been reported in the Oriental region and the Atlantic littoral where BG and SD sheep are dominant.

Serology showed a significant antibody response in all groups. Although goats are clinically more susceptible than sheep, sheep responded by a higher level of seroconversion than goats (Fig. 3) as has been found in other studies [20, 21].

The sheep and goat breeds used in this experiment included the most prevalent breeds in Morocco, representing almost 80% of sheep and 65% of goats. Sheep rarely develop clinical signs after infection with PPRV, but field experience from outbreaks of PPR in Morocco has indicated that young animals in intensive breeding systems may show clinical signs.

## Conclusions

This study revealed that significant clinical signs mainly occurred in goats of local Moroccan origin and TM sheep showing that surveillance based on development of clinical disease should focus on these breeds. However, the TM breed represents only 1/3 of the sheep population in the Atlas Mountains, which challenges an effective clinical surveillance. Surveillance based on serological screening is therefore recommended, especially in the coastline and Eastern plateau of Morocco, where breeds that are more resistant are the most prevalent. A control program on PPR based on vaccination should include sheep to avoid a reservoir for continuous spread of PPRV in Morocco.

## Abbreviations

BG: Beni Guil
BSL3: biosafety level 3
ELISA: enzyme-linked immunosorbent assay
FAO: Food and Agriculture Organization
IN: intra-nasal
IV: intravenous
OIE: World Organization for Animal Health
PPR: peste des petits ruminants
PPRV: peste des petits ruminants virus
RT-qPCR: real-time reverse transcriptase-polymerase chain reaction
SD: Sardi
TM: Timahdit
VN: virus neutralization
